# LncRNA IMFNCR Promotes Intramuscular Adipocyte Differentiation by Sponging miR-128-3p and miR-27b-3p

**DOI:** 10.3389/fgene.2019.00042

**Published:** 2019-02-11

**Authors:** Meng Zhang, Fang Li, Jun-wei Sun, Dong-hua Li, Wen-ting Li, Rui-rui Jiang, Zhuan-jian Li, Xiao-jun Liu, Rui-li Han, Guo-xi Li, Yan-bin Wang, Ya-dong Tian, Xiang-tao Kang, Gui-rong Sun

**Affiliations:** ^1^College of Animal Science and Veterinary Medicine, Henan Agricultural University, Zhengzhou, China; ^2^Henan Innovative Engineering Research Center of Poultry Germplasm Resource, Zhengzhou, China; ^3^The First Bethune Hospital, Jilin University, Changchun, China

**Keywords:** long non-coding RNA, ceRNA, miRNA, intramuscular adipocyte differentiation, chicken

## Abstract

Poultry meat quality is affected by many factors, among which intramuscular fat (IMF) is predominant. IMF content affects the tenderness, juiciness, and flavor of chicken. An increasing number of studies are focusing on the functions of lncRNAs in adipocyte differentiation. However, little is known about lncRNAs associated with intramuscular adipocyte differentiation. In the present study, we focused on an up-regulated lncRNA during intramuscular adipogenetic differentiation, which we named intramuscular fat-associated long non-coding RNA (IMFNCR). IMFNCR promotes intramuscular adipocyte differentiation. In-depth analyses showed that IMFNCR acts as a molecular sponge for miR-128-3p and miR-27b-3p and that PPARG is a direct target of miR-128-3p and miR-27b-3p in chicken. High-fat and high-protein diet inhibited chicken IMFNCR level *in vivo*. Moreover, IMFNCR level was positively correlated with PPARG mRNA level in chicken breast muscle tissues, a vital corollary to ceRNA function. Altogether, our research showed that IMFNCR acts as a ceRNA to sequester miR-128-3p and miR-27b-3p, leading to heightened PPARG expression, and thus promotes intramuscular adipocyte differentiation. Taken together, our findings may contribute to a more thorough understanding of chicken IMF deposition and the improvement of poultry meat quality.

## Introduction

Intramuscular fat (IMF) content is an important factor associated with meat quality, while abdominal fat is regarded as one of the main factors affecting poultry slaughter efficiency. It is generally believed that increasing IMF content is usually accompanied by an increase in abdominal fat content. Therefore, achieving carcass with high IMF content and minimal abdominal fat content, balancing IMF, and abdominal fat content has been a major aim of poultry breeding. Previous studies suggested that there exist different fat deposition rates in different parts of the body, and different fat tissues have distinct lipid accumulation and metabolism ability. However, the molecular mechanisms underlying depot-specific fat deposition remains largely unknown.

Peroxisome proliferator-activated receptor gamma (PPARG) is a critical transcriptional factor for regulating preadipocyte differentiation and lipid metabolism. PPARG is induced during the entire terminal differentiation process and is required for the activation of a number of adipogenic genes (Rosen et al., 2002). MicroRNAs are an important class of small non-coding RNAs that emerging as a post-transcriptional regulator involving different physiological activities such as growth, development and metabolism. Previous studies found that miR-27 increasing the beige/brown fat mass as a central upstream regulator of the transcriptional network involved in beige and brown adipogenesis after cold exposure (Karbiener et al., 2009; Lin et al., 2009; Lei and Mirko, 2014). Qiu et al. (2013) found that miR-128a inhibit the differentiation of pre-adipocyte in rat via regulating the expression of PPARG. Chen et al. (2018) demonstrated that miR-128-3p impeded preadipocyte differentiation and lipolysis by targeting PPARG and Sertad2 in 3T3-L1.

lncRNAs are a class of non-protein coding transcripts that are greater than 200 nt in length and found to be involved in various biological processes, such as cell cycle, cell differentiation, cell proliferation, and genomic imprinting (Koerner et al., 2009; Cesana et al., 2011; Batista and Chang, 2013; Tripathi et al., 2013). Recently, a growing number of reports have indicated that lncRNAs can communicate with and regulate protein-coding genes by direct competition for miRNA binding, thus acting as competing endogenous RNAs (ceRNAs) (Salmena et al., 2011; Legnini et al., 2014). Furthermore, it is now becoming evident that lncRNAs are emerging as critical regulators of skeletal muscle formation and fat deposition (Sun et al., 2013; Mueller et al., 2015). Linc-MD1, a muscle-specific long non-coding RNA was found to be regulating myogenesis by acting a sponge for miR-133 and miR-135 (Legnini et al., 2014). Sun et al. (2016) reported that a myoblast differentiation-associated long non-coding RNA, lncMD acts as a molecular sponge for miR-125b and that IGF2 is a direct target of miR-125b in cattle. Li et al. (2016) found adipocyte differentiation-associated long non-coding RNA (ADNCR) was downregulated during the differentiation of adipocyte and inhibited adipocyte differentiation by functioning as a competing endogenous RNA for miR-204. These individual studies demonstrate the growing importance of lncRNAs in myogenesis and adipogenesis. However, our knowledge of lncRNAs related to intramuscular adipocyte differentiation still remains limited.

Our previous transcriptome data of breast muscle at different physiological periods identified a lot of differentially expressed lncRNAs in breast muscle between juvenile and later laying-period hens (Zhang et al., 2017). A lncRNA, ALDB00000918, which we named IMF associated long non-coding RNA (IMFNCR), was found to be significantly upregulated in later laying-period hens. To investigate the potential function of lncRNAs associated with chicken IMF deposition, we further characterized the function and regulation mechanism of IMFNCR. Mechanistically, IMFNCR promotes intramuscular adipocyte differentiation by sponging miR-128-3p and miR-27b-3p, thereby augmenting the expression of the miR-128-3p and miR-27b-3p target gene, PPARG, which cooperates with C/EBPA and a number of adipogenic genes to execute the adipogenic differentiation program. Our findings provide new insights into understanding the mechanisms of IMF deposition and improvement of the meat quality of poultry.

## Materials and Methods

### Ethics Statement

All animal experiments were carried out in accordance with the Guidelines for Experimental Animals established by the Ministry of Agricultural of China (Beijing, China). This study was approved by the Animal Welfare Committee of College of Animal Science and Veterinary Medicine, Henan Agricultural University (Zhengzhou, Henan, China) (Permit Number:11-0085; Date: 06-2011).

### Animals and Tissue Collection

Eighteen Gushi chickens were divided into three groups: Low-fat diet group (Linoleic acid, 5%), middle-fat diet group (Linoleic acid, 10%), and High-fat diet group (Linoleic acid, 20%); another eighteen Gushi chickens were divided into three groups: Low-protein diet group (Crude protein, 10%), Middle-protein diet group (Crude protein, 20%) and High-protein diet group (Crude protein, 30%). All birds were raised in the same environmental conditions with *ad libitum* water and food for 3 weeks. Chickens were weighed and then killed by stunning and exsanguination 12 h after feed was withheld. Tissues were immediately collected, snap-frozen in liquid nitrogen and stored at −80°C until RNA extraction.

### Isolation, Culture of Primary Preadipocyte and Adipogenic Differentiation

**FIGURE 1:**
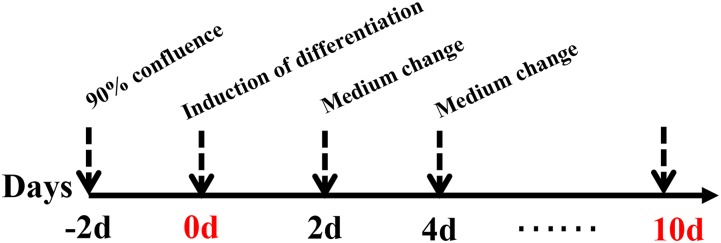
Induction of differentiation in intramuscular and abdominal preadipocyte. The basic medium consisted of DMEM/F-12 and 10% FBS. The induction differentiation medium consisted of basic medium, insulin, dexamethasone, 3-isobutyl-1-methylxanthine, and oleate. The maintenance medium consisted of basic medium, insulin, and oleate. The induction differentiation medium was replaced with the maintenance medium at 48 h, whereas the maintenance medium was replaced with basic medium at 96 h.

preadipocyte were isolated from the breast muscle and abdominal adipose tissue of female chickens at 2-week-old following methods described previously described (Ramsay and Rosebrough, 2003; Zhang et al., 2018). Cells were maintained in DMEM/F12 (1:1) supplemented with 10% FBS (Gibco, Beijing, China) and 1% penicillin/streptomycin solution in a humidified atmosphere with 5% (v/v) CO_2_ at 37°C. After cells reached confluence, differentiation was induced with differentiation medium [0.5 mM 3-isobutyl-1-methylxanthine, 1 μM dexamethasone, 50 nM insulin and 300 μM oleate (dissolved in DMSO) (all from Sigma, Beijing, China)] for 48 h. Then, the differentiation medium was replaced with maintenance medium [50 nM insulin and 300 μM oleate (Sigma)] and incubated for 48 h. The detailed procedure for the induction of intramuscular preadipocyte is described in Figure 1. Cells were harvested at 0, 2, 4, 6, 8, and 10 days after induction. Each stage included three biological replicates (*n* = 3).

### Plasmid Construction and Cell Transfection

The wild type and mutated sequences of IMFNCR and 3′UTR of *PPARG* (perfected the seed region of the miR-128-3p binding sites) were cloned into the XhoI–NotI site of the psiCHECK-2 (Promega, Maddison, WI, United States). The mutated sequences of IMFNCR and 3′UTR of *PPARG* were generated by mutating the seed region of the miR-128-3p binding sites by overlapping PCR. The siRNAs of IMFNCR were:IMFNCR-si1, 5′ GCUCUGGUCAAACACGCUUTT 3′, IMFNCR-si1, 5′ AAGCGUGUUUGACCAGAGCTT 3′; IMFNCR-si2, 5′ GCUAUAGAACGUCAGAAAUTT 3′ and IMFNCR-si2, 5′ AUUUCUGACGUUCUAUAGCTT 3′. miR-128-3p and miR-27b-3p mimics, inhibitor and negative control were purchase from GenePharma (Shanghai, China). Plasmid DNA was sequenced by Sangon Biotech (Shanghai, China) and extracted using an EndoFree Maxi Plasmid Kit (TIANGEN, Beijing, China). DF1 cells were cultured in DMEM with 10% fetal bovine serum (FBS) and 1% penicillin/streptomycin solution at 37°C with 5% CO_2_ in a humidified incubator.

### Luciferase Assays

DF1 cells were seeded in 6-well plates at a density of 5 × 10^5^ cells/well and cultured under routine conditions with 10% FBS. When the cells reached 70 or 80% confluence, the IMFNCR wild-type or mutant construct was cotransfected with 50 nM negative control or miR-128-3p mimic (GenePharma, Shanghai, China) using Kretschmer-Kazemi and Sczakiel (2003) (Invitrogen, Carlsbad, CA, United States) according to the manufacturer’s instructions, and the medium was replaced 6 h later. The relative luciferase activity was measured 48 h after transfection by the Dual-Luciferase Reporter Assay System (Promega) on a Fluoroskan Ascent FL instrument (Thermo Fisher Scientific, Shanghai, China). Renilla luciferase activity was normalized to firefly luciferase activity.

### RNA Isolation and Real-Time Quantitative PCR (qPCR)

Total RNA from tissues and preadipocyte were isolated using extracted with Trizol reagent according to the manufacturer’s protocol (Takara, Dalian, China). RNA samples were stored at −80 C until used. cDNA synthesis and qPCR were carried out as described (Zhang et al., 2017, 2018). qPCR primers are reported in Supplementary Table S1. The expression of miRNA was detected by stem-loop real-time qPCR. The stem-loop primers used for the qPCR, miRNA mimics, miRNA inhibitor and negative control were purchased from GenePharma Co., Ltd. (Shanghai, China). We used the 2^-ΔΔCt^ method to analyze relative expression levels of mRNA, lncRNA and miRNA.

### Western Blot Analysis

Total protein was extracted from cells using a RIPA buffer (Solarbio) supplemented with PMSF (Servicebio) (100:1). Protein was separated on 10% SDS-PAGE gels. The proteins were transferred to PVDF membranesm, and then blocked with 5% non-fat milk for 2 h. The membranes were washed with PBST three times (5 min/time) and incubated with the primary antibodies (Abcam) at 4°C for overnight. Then the membranes were washed three times using PBST and incubated with secondary antibody conjugated with HRP (Abcam) for 1 h at room temperature. Signals were detected by ECL Plus (Solarbio). β-*Actin* was used as an internal control.

### RNA Fluorescence *in situ* Hybridization (RNA FISH)

FITC-labeled IMFNCR probes were obtained from servicebio (Wuhan, Hubei, China). RNA FISH was performed using fluorescent *in situ* hybridization kit (RiboBio) following the manufacturer’s instructions. The cytopalsmic and nuclear RNA were isolated by PARIS^TM^ Kit (Life) according to the manufacturer’s protocol.

### Oil Red O Staining

Cells were washed with PBS three times and fixed with 10% formaldehyde for 30 min. The fixed cells were washed with PBS three times and incubated with 1% filtered Oil Red O solution (0.3% Oil Red O, 60% isopropanol, and 40% PBS) for 30 min, and then phase-contrast microscope (Olympus, Japan) was used to check for positive cells appearing red. Subsequently, Oil Red O was eluted from the stained cells with 100% isopropanol (v/v) and quantified by microplate reader (Thermo Fisher Scientific) at 510 nm.

### Statistical Analysis

Data were analyzed using Student’s tests in SPSS 20.0 software. Data are presented as the mean ± SEM (standard error of the mean) based on at least three replicates. Correlation analysis was conducted using Pearson of bivariate correlation. A *p*-value of <0.05 was considered significant.

## Results

### Identification of IMFNCR as a Candidate lncRNA

**FIGURE 2:**
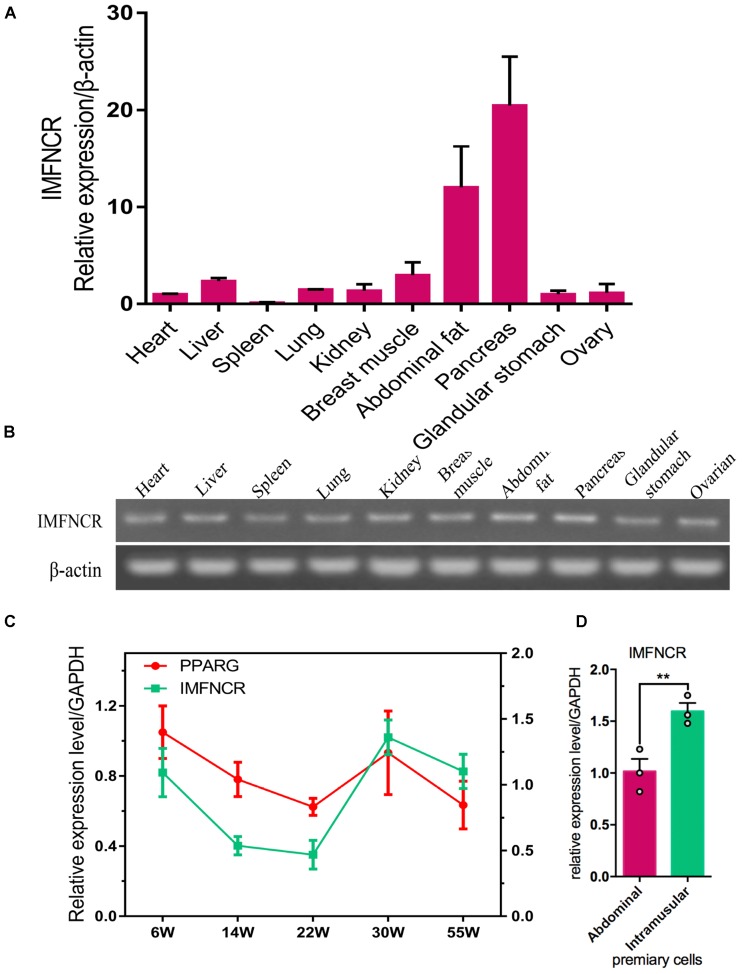
Characteristics of chicken IMFNCR in different tissues and cells. **(A)** Tissue expression profile of IMFNCR by qRT-PCR. **(B)** Tissue expression profile of IMFNCR by semi-quantitative PCR. **(C)** The expression dynamics of IMFNCR in breast muscle tissues at different physiological periods. **(D)** The expression level of IMFNCR in abdominal and intramuscular preadipocyte. Data represent means ± SEM (*n* = 3). ^∗^*p* ≤ 0.05; ^∗∗^*p* ≤ 0.01.

According to our previous mRNA and lncRNA sequencing data (NCBI Project No: PRJNA380024), a candidate intramuscular fat-associated long non-coding RNA (IMFNCR) was remarkably up-regulated in chicken breast muscle at later-laying period compared with that at pre-laying period. The genomic location and gene structure of IMFNCR are shown in Supplementary Figure S1. qRT-PCR results showed that IMFNCR was highly expressed in pancreas, abdominal fat and breast muscle tissues (Figure 2A,B) and the expression trend of IMFNCR was consisted with PPARG in breast muscle at different periods (Figure 2C). It is interesting to note that IMFNCR were highly expressed in intramuscular adipocyte compared with abdominal adipocyte (Figure 2D), implying their potential biological functions in chicken IMF deposition.

**FIGURE 3:**
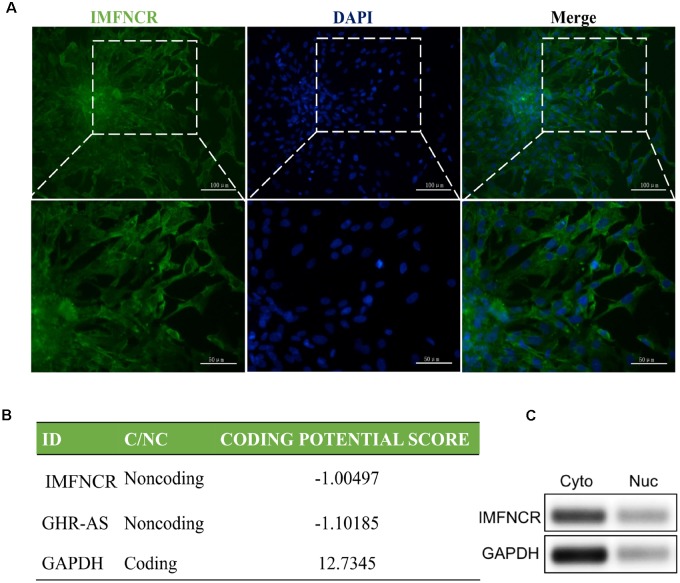
Subcellular localization and encoding ability prediction of IMFNCR. **(A)** Intramuscular preadipocyte were stained with FITC-labeled IMFNCR probes and visualized by fluorescence. **(B)** The RNA sequences of IMFNCR, GHR-AS, and GAPDH were put into the Coding Potential Calculator (CPC) program, and both IMFNCR and GHR-AS were predicted to be non-coding RNAs, while GAPDH was identified to code for protein. **(C)** IMFNCR is mainly localized in the cytoplasm of intramuscular preadipocyte. RNA isolated from cytoplasm (Cyto) and nuclear (Nuc) fractions of preadipocyte and adipocyte was used to analyze the expression level of IMFNCR by semi-quantitative PCR. GAPDH mRNA was used as control.

RNA FISH and semi-quantitative PCR of nuclear and cytoplasmic fractions analyses indicated that IMFNCR is predominantly localized in the cytoplasm of preadipocyte (Figure 3A,C). To assess whether IMFNCR has protein coding ability, online software was used in the present study. CPC (Coding Potential Calculator) software showed that IMFNCR has a very low coding potential, similar to GHR-AS, a well-known lncRNA (Zhang et al., 2016; Figure 3B).

### IMFNCR Promotes Intramuscular Adipocyte Differentiation

**FIGURE 4:**
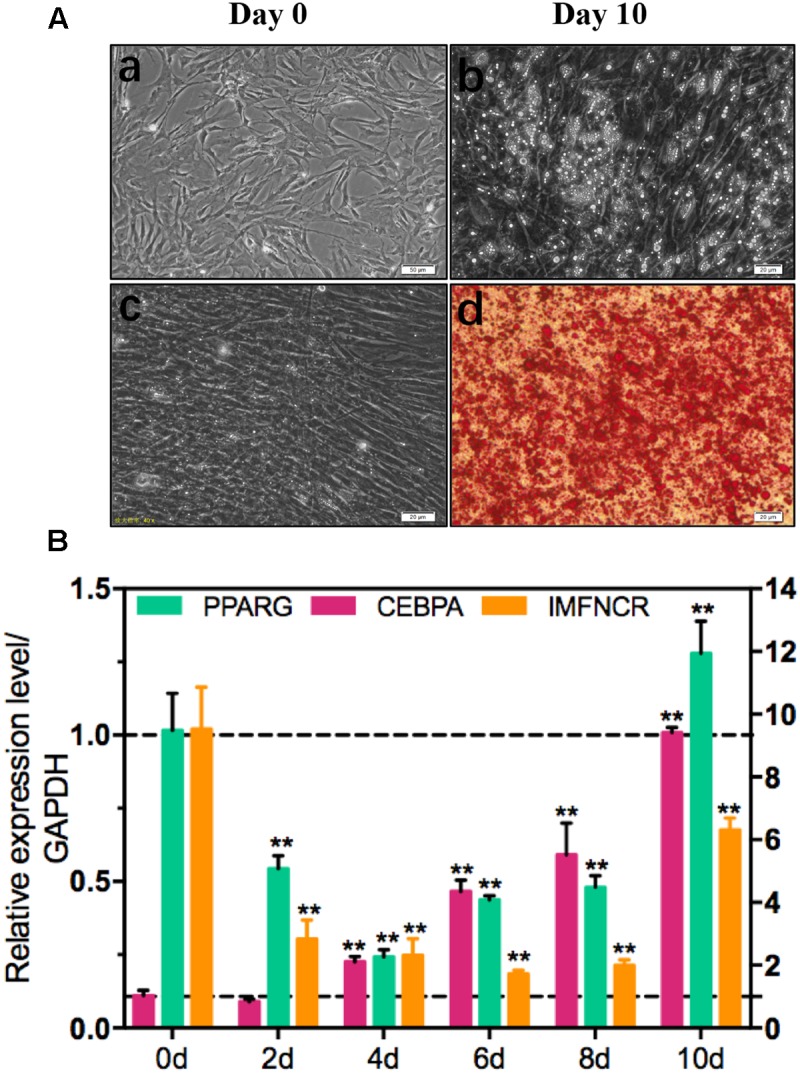
Induction of chicken intramuscular preadipocyte differentiation. Oil Red O staining **(A)** and qRT-PCR **(B)** analysis of IMFNCR and adipogenic markers, PPARG, and CEBPA confirm the identity of chicken intramuscular preadipocyte. Gene expression is plotted as fold-change relative to day 0 (mean ± SEM, *n* = 3, ^∗^*p* ≤ 0.05, ^∗∗^*p* ≤ 0.01).

**FIGURE 5:**
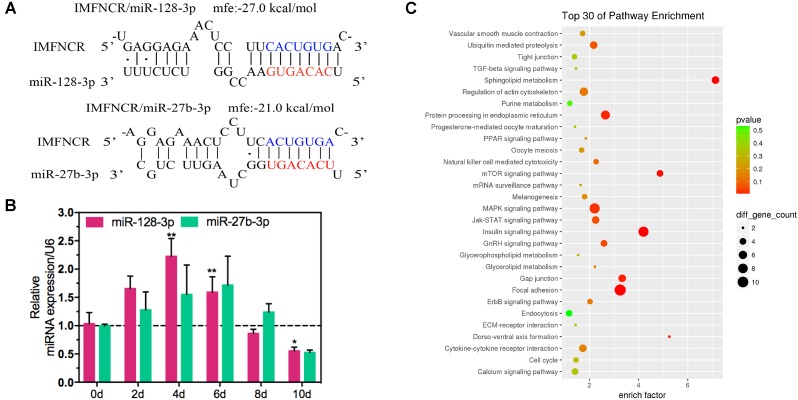
lncRNA IMFNCR was a potential target gene of miR-128-3p and miR-27b-3p. **(A)** RNA hybrid predicted miR-128-3p and miR-27b-3p binding sites in IMFNCR. Mfe, minimal free energy (kcal/mol). Nucleotides of the miR-128-3p and miR-27b-3p seed region (positions 2–8) are marked in red. Mutated nucleotides are in blue. **(B)** Expression pattern of miR-128-3p and miR-27b-3p during chicken intramuscular preadipocyte differentiation. **(C)** KEGG pathway analysis of intersection target genes of miR-128-3p and miR-27b-3p. (mean ± SEM, *n* = 6, ^∗^*p* ≤ 0.05, ^∗∗^*p* ≤ 0.01).

To explore the function of IMFNCR in IMF deposition, we constructed intramuscular adipocyte differentiation model according to our previous research (Zhang et al., 2018). After inducing with adipogenic agents for 10 days, chicken intramuscular adipocyte was fully differentiated and filled with large lipid droplets (Figure 4A). QPCR results suggested that IMFNCR and adipocyte markers PPARG and CEBPA were significantly upregulated with adipocyte differentiation (Figure 4B). miR-27b-3p has been identified to participate in adipocyte differentiation (Lin et al., 2009). Interestingly, we noticed that miR-128-3p share almost the same seed region with miR-27b-3p, which indicates that they may target the same genes. There are 322 intersection genes between miR-128-3p and miR-27b-3p. Bioinformatics analysis showed that there exist a putative miR-128-3p and miR-27b-3p binding site in IMFNCR sequence (Figure 5A). qRT-PCR result showed that miR-128-3p and miR-27b-3p were significantly downregulated, while IMFNCR and PPARG were significantly upregulated with intramuscular adipocyte differentiation (Figure 5B). Furthermore, we noticed that those target genes were mainly enriched in Insulin signaling pathway, MAPK signaling pathway, Focal adhesion, and PPARG signaling pathway (Figure 5C).

**FIGURE 6:**
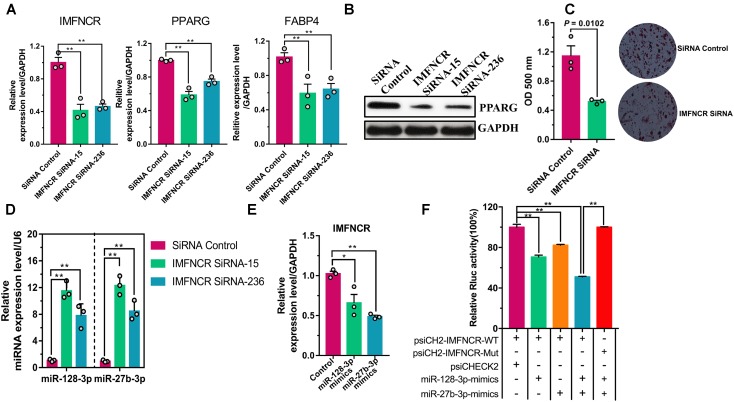
lncRNA IMFNCR functions as a miRNA sponge. **(A)** IMFNCR knockdown decreased PPARG and FABP4 expression. **(B)** IMFNCR knockdown inhibited intramuscular adipogenesis, as indicated by Oil Red O staining and quantified by microplate reader at 510 nm. **(C)** IMFNCR knockdown inhibited the protein expression of PPARG. **(D)** IMFNCR knockdown increased the expression of miR-128-3p and miR-27b-3p. **(E)** miR-128-3p and miR-27b-3p inhibited the expression of IMFNCR. **(F)** miR-128-3p and miRNA mixture overexpression inhibited Rluc expression of the Rluc activity of psiCH2-IMFNCR-WT, while psiCH2-IMFNCR-WT-Mut no longer responded to miRNA mixture (miR-128-3p and miR-27b-3p). MiR-128-3p and miR-27b-3p mimics were transfected into DF1 cells, together with psiCH2-IMFNCR-WT, or psiCH2-IMFNCR-WT-Mut. The data represent means ± SEM (*n* = 3). ^∗^*p* ≤ 0.05; ^∗∗^*p* ≤ 0.01.

To better understand the role of IMFNCR function, we performed knockdown IMFNCR in intramuscular adipocyte (Figure 6A). Our result showed that siRNAs specific to IMFNCR reduced its RNA level by 40 and 44%, and the expression of adipogenic markers PPARG and FABP4 decreased significantly (Figure 6A,B). Moreover, Oil red O staining suggested that knockdown of IMFNCR inhibited adipogenesis (Figure 6C). Furthermore, miR-128-3p and miR-27b-3p was significantly upregulated in IMFNCR knockdown group compared with control group (Figure 6D). When we overexpressed miR-128-3p or miR-27b-3p, the expression level of IMFNCR decreased significantly (Figure 6E). We observed that miR-128-3p or miR-27b-3p and both miR-128-3p and miR-27b-3p significantly reduced the Rluc activity of the wild-type IMFNCR reporter vector (psiCH2-IMFNCR-WT) in DF1 cells, respectively (Figure 6F).

### miR-128-3p and miR-27b-3p Inhibits Adipogenesis by Targeting PPARG

**FIGURE 7:**
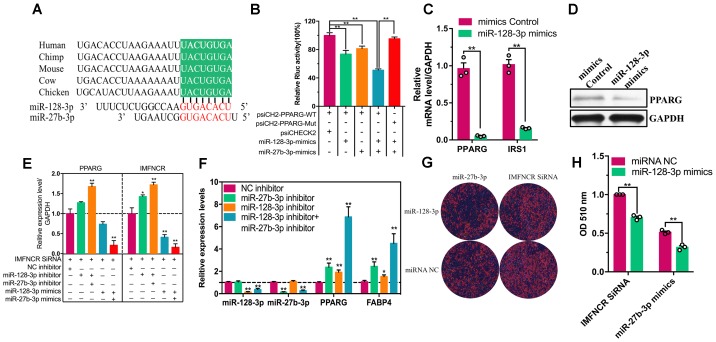
IMFNCR promotes intramuscular adipocyte differentiation by sponging miR-128-3p and miR-27b-3p. **(A)** The putative miR-128-3p and miR-27b-3p-binding sites at PPARG 3′UTR (green) are evolutionarily conserved across species. **(B)** miR-128-3p and miR-27b-3p suppresses PPARG translation. miR-128-3p and miR-27b-3p were transfected into DF1 cells, along with PPARG-UTR-WT but not PPARG-UTR-Mut. The luciferase activity was analyzed 48 h later. **(C)** miR-128-3p suppresses the expression of PPARG and IRS1. Intramuscular preadipocyte was infected with miR-128-3p mimics or mimics Control at 37°C, followed by the addition of fresh growth medium. RNA and protein were extracted 48 h later, and levels were accessed by qRT-PCR and **(D)** western blot. **(E)** IMFNCR siRNA and inhibitor (miR-128-3p inhibitor, mixture inhibitor) or mimics (miR-128-3p mimics, mixture mimics) were transfected into chicken intramuscular preadipocyte. qRT-PCR assays were performed to determine the expression levels of PPARG and IMFNCR. **(F)** Negative control inhibitor (NC inhibitor) or miR-128-3p or mixture (miR-128-3p and miR-27b-3p) inhibitor was transfected into chicken intramuscular preadipocyte. Forty-eight hours later, qRT-PCR assays were performed to determine the expression levels of miR-128-3p, miR-27b-3p, PPARG, and FABP4. **(G)** IMFNCR siRNA and miR-27b-3p were transfected into miRNA mimics NC or miR-128-3p mimics. At 48 h intracellular triglyceride content was measured by Oil Red O staining and **(H)** quantified by microplate reader at 510 nm. The data represent means ± SEM (*n* = 3). ^∗^*p* ≤ 0.05; ^∗∗^*p* ≤ 0.01.

In search of potential downstream effectors of IMFNCR-miR-128-3p/miR-27b-3p mediated regulation of intramuscular adipocyte differentiation, we focused on PPARG, a major regulator of adipogenic genes. Bioinformatics analysis for miRNA recognition sequences on chicken PPARG also revealed the presence of a putative miR-128-3p and miR-27b-3p binding site (Figure 7A). To verify the correlation between PPARG and miR-128-3p and miR-27b-3p in chicken, we fused the 3′UTR of chicken PPARG to a luciferase reporter gene. A remarkable repression of Rluc activity of psiCHECK2-PPARG-WT reporter was observed in DF1 cells transfected with miR-128-3p and miR-27b-3p, whereas, the Rluc activity of psiCHECK2-PPARG-WT transfected with both miR-128-3p and miR-27b-3p mimics group was significantly reduced comparing with psiCHECK2-PPARG-Mut group (Figure 7B). In addition, overexpressed and knockdown miR-128-3p altered PPARG and IRS1 (a well-known target gene of miR-128-3p) expression levels in chicken intramuscular adipocyte (Figure 7C,D). These results suggested that PPARG is indeed a direct target of miR-128-3p and miR-27b-3p in chicken.

### IMFNCR Increases the Expression of PPARG in a miR-128-3p and miR-27b-3p Dependent Manner

To confirm that the observed effects of IMFNCR were due to miR-128-3p and miR-27b-3p-mediated regulation of PPARG, we transfected miR-128-3p and miR-27b-3p mimics or inhibitor into chicken intramuscular preadipocyte. As expected, miR-128-3p overexpression strongly decreased the mRNA levels of IMFNCR and PPARG and inhibited adipogenesis (Figure 6E, 7G,H). In a reciprocal experiment, increased mRNA levels of both IMFNCR and PPARG were observed in miR-128-3p inhibitor and mix (mixed miR-128-3p and miR-27b-3p inhibitor-treated cells) group (Figure 7E). As shown in Figure 7F, miR-27b-3p and miR-128-3p inhibitor increased the mRNA levels of adipogenic markers (PPARG and FABP4). These results agreed with the ceRNA hypothesis that IMFNCR inhibits miR-128-3p and miR-27b-3p function, leading to derepression of their target gene PPARG.

### Effects of Dietary Nutrient Level on IMFNCR Expression

**FIGURE 8:**
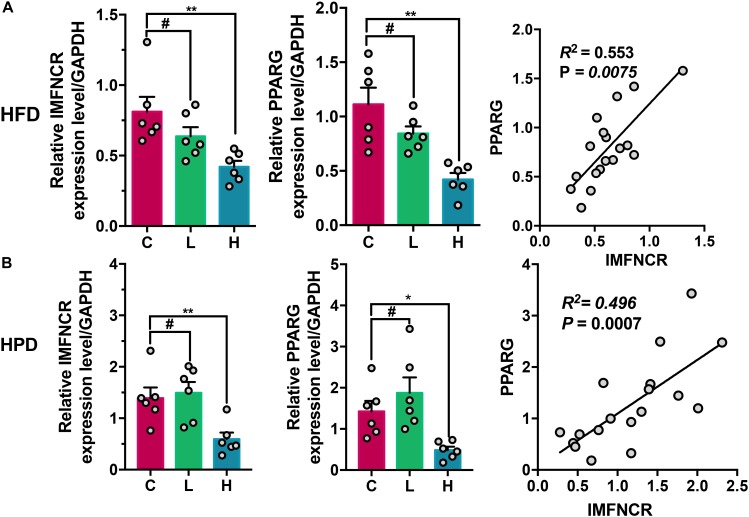
Effects of dietary nutrient level on IMFNCR expression. **(A)** HFD inhibits IMFNCR expression in breast muscle tissues. The breast muscle tissues of chicken were collected after 3-weeks feeding (C, control diet group; L, low-fat diet group; H, high-fat diet group). **(B)** HPD inhibits IMFNCR expression in breast muscle tissues. The breast muscle tissues of chicken were collected after 3-weeks feeding (C, control diet group; L, low-protein diet group; H, high-protein diet group). Data are presented as means ± SEM, *n* = 6, ^∗^*p* ≤ 0.05, ^∗∗^*p* ≤ 0.01. ^#^No significant difference.

In order to understand the effect of different nutrient levels on miRNA expression, chicken was fed with high fat (HFD) and high protein diet (HPD) for 2 weeks. As indicated in Figure 8A,B, the expression level of IMFNCR and PPARG were significantly decreased in both HFD and FPD groups (*P* < 0.01) (Figure 8A,B). Moreover, a significantly positive correlation of expression between IMFNCR and PPARG was observed in chicken breast muscle tissues (*P* = 0.0075 and *P* = 0.0007, respectively) (Figure 8).

**FIGURE 9:**
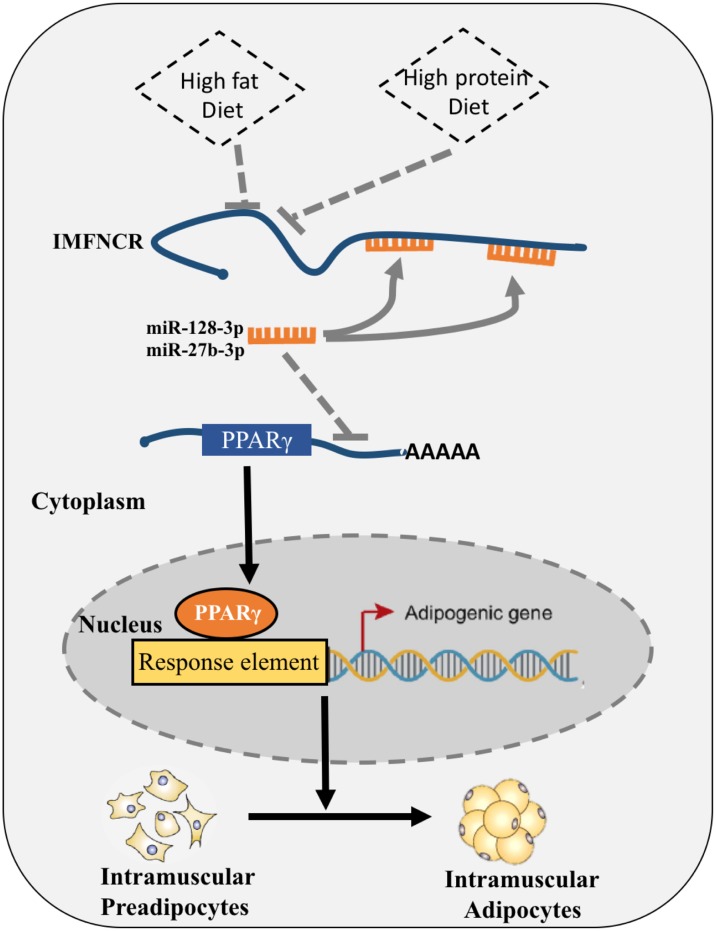
Proposed model of IMFNCR regulation on intramuscular adipocyte differentiation. High-protein and High-fat diet inhibit the expression of IMFNCR; IMFNCR promotes intramuscular adipogenesis differentiation by functioning as a ceRNA, which sequesters miR-128-3p and miR-27b-3p, thereby protecting PPARG transcripts from miRNA-mediated suppression in chicken.

Taken together, these results strongly suggest that IMFNCR, which was regulated by nutrient factor and by binding both miR-128-3p and miR-27b-3p, acts as a decoy to abolish miRNA repressing activity on PPARG and thereby promotes chicken intramuscular differentiation (Figure 9).

## Discussion

Meat quality is affected by many factors, among which IMF content contributes to the tenderness, juiciness and flavor of the meat (Aaslyng et al., 2003). IMF is mainly deposited in the muscle mass with fat, distributed in the myometrium, myofibrils, and endometrium, as well as in the form of lipid droplets in the muscle cytoplasm, which includes intracellular phospholipid, triglyceride, and cholesterol (Qiu et al., 2017; Zhang et al., 2018). IMF content is an important quantitative trait, which is affected by both nutritional and genetic factors, such as mRNA, DNA methylation and miRNAs (Gerbens et al., 2001; Cui et al., 2012; Chen et al., 2017; Du et al., 2018). Our previous studies have found that the late-laying-period hens exhibited a higher IMF content in breast muscle tissues, moreover, some miRNAs regulated mRNAs to affect IMF content (Zhang et al., 2017, 2018). Several lncRNAs have been identified and suggested to regulate adipogenesis, such as steroid receptor RNA Activator (SRA) (Xu et al., 2010), adipogenic differentiation induced non-coding RNA (ADINR) (Xiao et al., 2015) and adipocyte differentiation-associated long non-coding RNA (ADNCR) (Li et al., 2016). These individual researches demonstrate the growing importance of lncRNAs in adipogenesis, but our knowledge of lncRNAs related to intramuscular adipocyte differentiation still remains limited.

In the present study, we reanalyzed the transcriptome data of chicken breast muscle tissue at different physiological periods. Further analysis revealed a large number of differentially expressed lncRNAs among different development stages, including IMFNCR, a novel fat-specific lncRNA that can promote adipogenesis in primary chicken intramuscular adipocyte. Furthermore, we found IMFNCR promotes chicken intramuscular adipocyte differentiation via binding miR-128-3p and miR-27b-3p.

LncRNAs have been suggested to function as miRNA sponges, however, only a few of such lncRNAs have been reported to have functions in adipogenesis. For instance, lncRNA ADNCR acts as a ceRNA by sponging miR-204 thereby protecting SIRT1 transcripts in bovine Adipocyte-derived stem cells (ADSCs) (Li et al., 2016). Here we provide direct evidence that IMFNCR can function as a ceRNA by sponging miR-128-3p and miR-27b-3p, thereby protecting PPARG transcripts from miR-128-3p- and miR-27-3p-mediated suppression (Figure 9). IMFNCR RNAi and miRNA (miR-128-3p, miR-27b-3p or mix miR-128-3p and miR-27b-3p) overexpression in chicken intramuscular preadipocyte produced a similar decrease in the expression of adipogenic markers (PPARG and FABP4), whereas IMFNCR RNAi and miRNA (miR-128-3p, miR-27b-3p or mix miR-128-3p and miR-27b-3p) knockdown led to increased adipogenic differentiation.

In search of potential downstream effector of IMFNCR/miR-128-3p and miR-27b-3p mediated regulation of intramuscular adipocyte differentiation, we focused on PPARG, a major regulator of adipogenic differentiation (Nedergaard et al., 2005; Bai et al., 2007). Wang et al. (2011) found that miR-27a suppressed porcine adipocyte differentiation. Kim et al. (2010) found that miR-27a would suppress adipocyte differentiation through targeting PPARG. Qiu et al. (2013) also found that miR-128a inhibit the differentiation of pre-adipocyte in rat via regulating the expression of PPARG. In line with the results observed from rat pre-adipocyte, our experiments suggested that miR-128-3p and miR-27b-3p were negative regulator of chicken intramuscular adipocyte differentiation. Mutation of the seed region of the predicted miR-128-3p and miR-27b-3p target site abolished the regulation of the PPARG 3′UTR reporter, demonstrating that PPARG is a direct target of miR-128-3p and miR-27b-3p in chicken intramuscular adipocyte differentiation. Furthermore, we show that the observed effects of IMFNCR on adipogenic differentiation were due to miR-128-3p and miR-27b-3p mediated regulation of PPARG. IMFNCR modulates PPARG expression by competing for miR-128-3p and miR-27b-3p as a ceRNA to regulate intramuscular adipocyte differentiation.

Nutrients can regulate gene expression in the body’s activities at various levels and pathways, that is, nutrients affect the growth, development, and reproduction of animals (Hsu and Ding, 2003; Zhou et al., 2010). One of the ways is to influence gene expression. Tian et al. (2017) found that feeding low-protein diets can promote fatty acid transport and synthesis-related genes in the longissimus dorsi (LDM), but the deposition of IMF in pig LDM is significantly reduced. Several studies suggest that the addition of PUFAs diets inhibit fat accumulation and adipocyte differentiation (Madsen et al., 2005; Zhou et al., 2010; Wójcik et al., 2014). Hsu et al. demonstrated that polyunsaturated FA inhibit the ADD1 expression and involved in lipid metabolism in porcine adipocyte (Hsu and Ding, 2003). Wójcik et al. (2014) found that omega-3 polyunsaturated fatty acids (0309-3 PUFAs) decrease expression of SREBP1 while inducing expression of adipophilin and GLUT4. Our results suggested that high-fat diet decreased the expression levels of PPARG and IMFNCR in breast muscle tissues. Increasing the energy level in the feed increases the percentage of abdominal fat and the adiponectin gene expression abundance, while lowering the dietary protein level increases the percentage of abdominal fat and adiponectin gene expression in abdominal fat (Tahmoorespur et al., 2010). In the present study, we found that high-protein diet significantly decreased both PPARG and IMFNCR mRNA levels. Moreover, the IMFNCR RNA levels was significantly positively correlated with PPARG mRNA level between different nutrient level (*P* < 0.001). Our results indicated that lncRNA IMFNCR may contribute to molecular genetic selection to balance IMF and abdominal fat content in chicken breeding. In addition, IMFNCR may serve as epigenetic markers for evaluating meat quality in chicken and will be likely to contribute to the improvement of poultry meat quality.

In summary, our study provides the first evidence of lncRNAs which could have functional roles in chicken intramuscular adipocyte differentiation. Importantly, we combine nutrient factor with detailed mechanistic studies to describe an adipocyte-differentiation related lncRNA, IMFNCR, that functions as a ceRNA to promote intramuscular adipogenic differentiation. Our study provides a better understanding of researches underlying intramuscular adipogenic differentiation in poultry and may also contribute to improving poultry meat quality.

## Author Contributions

MZ, G-rS, and X-tK conceived of and designed the experiments. MZ, FL, and J-wS performed the experiments. MZ, D-hL, and G-xL analyzed the data. W-tL, Z-jL, X-jL, Y-bW, Y-dT, R-lH, and R-rJ contributed to reagents, materials, and analysis tools. MZ wrote the manuscript.

## Conflict of Interest Statement

The authors declare that the research was conducted in the absence of any commercial or financial relationships that could be construed as a potential conflict of interest.

## Supplementary Material

FIGURE S1 Characterization of the IMFNCR Sequence.

TABLE S1 Specific primers used for qRT-PCR.
