# Supplementary material for: LncRNA IMFNCR Promotes Intramuscular Adipocyte Differentiation by Sponging miR-128-3p and miR-27b-3p
Source: Front Genet. 2019 Feb 11;10:42. doi: 10.3389/fgene.2019.00042 (PMC6378276; doi:10.3389/fgene.2019.00042)
Supplement: FIGURE S1 — Characterization of the IMFNCR Sequence. [file Image_1.pdf]

LncRNA IMFNCR promotes intramuscular adipocytes differentiation by sponging miR-128-3p and miR-27b-3p

Meng Zhang, Fang Li, Jun-wei Sun, Dong-hua Li, Wen-ting Li, Rui-rui Jiang, Zhuan-jian Li, Xiao-jun Liu, Ruili Han, Guoxi Li, Yanbin Wang, Yadong Tian, Xiang-tao Kang and Gui-rong Sun*

[1]College of Animal Science and Veterinary Medicine, Henan Agricultural University, Zhengzhou, 450002, China.

[2]Henan Innovative Engineering Research Center of Poultry Germplasm Resource, Zhengzhou 450002, China.

Correspondence and requests for materials should be addressed to G.-R.S. (email: grsun2000@126.com)

Supplementary Figure S1

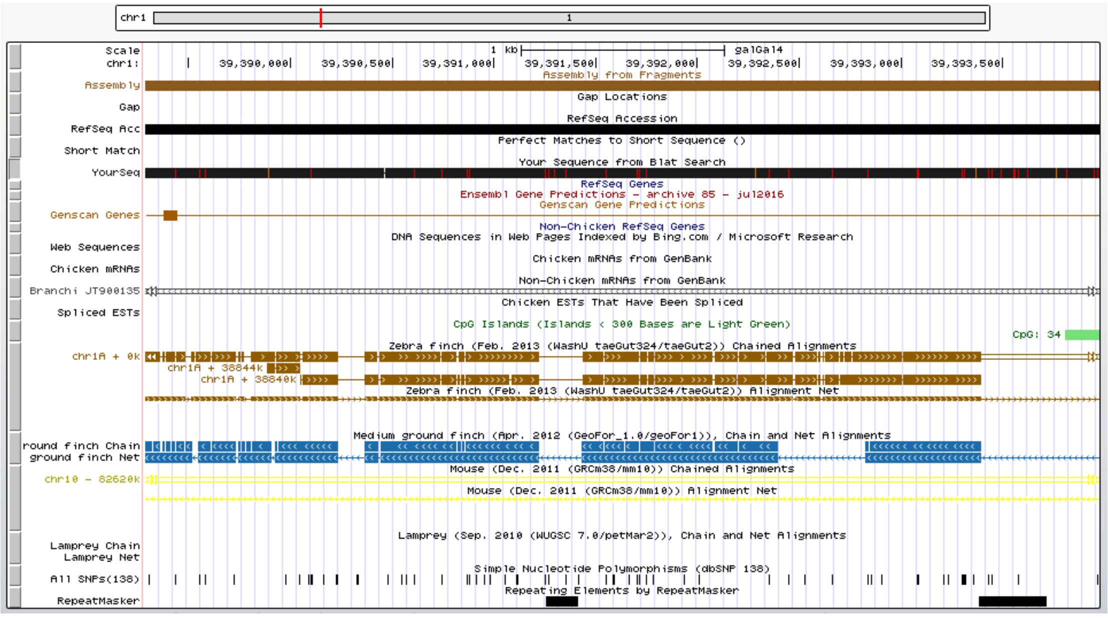


```
00000001 cacccgcgcccttcagaggagcgttcccccgtgtctctcggccttgctcg 00000050
<<<<<<<< |||||||||||| ||||||||||||||| ||||||||||||||||||||| <<<<<<<<
39393976 cacccgcgccctgcagaggagcgttccccggtgtctctcggccttgctcg 39393927

00000051 ggcccgtccgagccgcccctcagccggcggcgttcccaggccgcgcctga 00000100
<<<<<<<< |||||||||||||||||||||||||||||||||||||||||||||||||| <<<<<<<<
39393926 ggcccgtccgagccgcccctcagccggcggcgttcccaggccgcgcctga 39393877

00000101 cacctgccggcggccgcccgcgctgcgccacgcgagtcgcggcacagggc 00000150
<<<<<<<< |||||||||||||||||||||||||||||||||||||||||||||||||| <<<<<<<<
39393876 cacctgccggcggccgcccgcgctgcgccacgcgagtcgcggcacagggc 39393827

00000151 ctcggcgcttggaaaacgaatccctcttctgtgggattgttccatggacg 00000200
<<<<<<<< ||||||||||||||| |||||||||||||||||||||||||||||||||| <<<<<<<<
39393826 ctcggcgcttggaaagcgaatccctcttctgtgggattgttccatggacg 39393777

00000201 ttcaggagttttagtttttttttttctttcttttgtataaagtaggtaac 00000250
<<<<<<<< |||||||||||||| ||||||||||||||||||||||||||||||||||| <<<<<<<<
39393776 ttcaggagttttag.ttttttttttctttcttttgtataaagtaggtaac 39393728

00000251 gtattttaactgtaacgtggagaaaagaaggctgagaggggatcttatcg 00000300
<<<<<<<< |||||||||||||||||||||||||||||||||||||||||||||||||| <<<<<<<<
39393727 gtattttaactgtaacgtggagaaaagaaggctgagaggggatcttatcg 39393678
```

```
00000301 gtgctcgttaataaccaagtgtatggggccaggctctgttcagcggtgcc 00000350
<<<<<<<< |||||||||||||||||||||||||||||||||||||||||||||||||| <<<<<<<<
39393677 gtgctcgttaataaccaagtgtatggggccaggctctgttcagcggtgcc 39393628

00000351 cagtggcaggacaaggggcagtgagcacaaactggaatgtggaagttcca 00000400
<<<<<<<< ||| |||||||||||||||||||||||||||||||||||||||||||||| <<<<<<<<
39393627 cagcggcaggacaaggggcagtgagcacaaactggaatgtggaagttcca 39393578

00000401 tacgaacgtgaggaggagctcctgcactgtgacggtgacagagcactggg 00000450
<<<<<<<< || |||||||||||| | ||||| |||||||||||||||||||||||||| <<<<<<<<
39393577 tatgaacgtgaggagaaactccttcactgtgacggtgacagagcactggg 39393528

00000451 acaggctgcccagagaggctgtggagtctccttctgtggagatattcaaa 00000500
<<<<<<<< ||||||||||||||||||||||||||||||||||| |||||||||||||| <<<<<<<<
39393527 acaggctgcccagagaggctgtggagtctccttctatggagatattcaaa 39393478

00000501 atccacctggatgctttcctgtgagacctgcatcagagcagatgatctca 00000550
<<<<<<<< ||||||||||||||||||||||||||||||| |||||||||||||||||| <<<<<<<<
39393477 atccacctggatgctttcctgtgagacctgcgtcagagcagatgatctca 39393428

00000551 atgggaccctcctaacccctgaattctgtgtgattctgtgatttttaagt 00000600
<<<<<<<<  ||||||||||||||||||||||||||||||||||||||||||||||||| <<<<<<<<
39393427 gtgggaccctcctaacccctgaattctgtgtgattctgtgatttttaagt 39393378

00000601 gtattcagcaccatctcacttgccgctttgggacatctgtcgtgtagcca 00000650
<<<<<<<< ||||||||   ||||||||||||||||||||||||||||||||||||||| <<<<<<<<
39393377 gtattcag...catctcacttgccgctttgggacatctgtcgtgtagcca 39393331

00000651 caatacttcacagcaatagaggctatagaatgtcagaaatttaatttcct 00000700
<<<<<<<< |||||||||||||||||| ||||||||||| ||||||||||||||||||| <<<<<<<<
39393330 caatacttcacagcaatacaggctatagaacgtcagaaatttaatttcct 39393281

00000701 taagttaaacttagagcaatattttcctgcctctaacacttcccaggact 00000750
<<<<<<<< |||||||||||||||||||||||||||||||||||||||||||||||||| <<<<<<<<
39393280 taagttaaacttagagcaatattttcctgcctctaacacttcccaggact 39393231

00000751 ttactgagctgctgtgcatatacgaagctgtgggaagataggtatgtgtt 00000800
<<<<<<<< |||||||||||||||||||||||||||||||||||||||||||||||||| <<<<<<<<
39393230 ttactgagctgctgtgcatatacgaagctgtgggaagataggtatgtgtt 39393181

00000801 taatttaaaaaaggctgatggttgtgtttacaggaattacattctagtcc 00000850
<<<<<<<< |||||||||||||||||||||||||||||||||||||||||||||||||| <<<<<<<<
39393180 taatttaaaaaaggctgatggttgtgtttacaggaattacattctagtcc 39393131
```

```
00000851 ttcattcctctctgcattcctttcattcaggggctcagactgtttacatg 00000900
<<<<<<<< ||||||||||||||||||||||||||||||||||||||||||||||| || <<<<<<<<
39393130 ttcattcctctctgcattcctttcattcaggggctcagactgtttacttg 39393081

00000901 gtccgcttccctggagttacgctaaaccctaaaggacaacattttgaatt 00000950
<<<<<<<< ||| |||||||||||||||||||||||||||||||||||||||||||||| <<<<<<<<
39393080 gtctgcttccctggagttacgctaaaccctaaaggacaacattttgaatt 39393031

00000951 ctaaagaagtctccttgggaaactcctcccagatgaatggtctgtgtgca 00001000
<<<<<<<< |||||||||||||||||||||||||||||||||||||||||||||||||| <<<<<<<<
39393030 ctaaagaagtctccttgggaaactcctcccagatgaatggtctgtgtgca 39392981

00001001 gagctggtgggaaccaacagctttaaatagtcagcatcagcacaaaatgt 00001050
<<<<<<<< |||||||||||||||||||||||||||||||||||||||||||||||||| <<<<<<<<
39392980 gagctggtgggaaccaacagctttaaatagtcagcatcagcacaaaatgt 39392931

00001051 ttttcggtagtcctgaaaccagggctactgcctgtgctccaggtgcttgt 00001100
<<<<<<<< |||||||||||||||||||||||||||||||||||||||||||||||||| <<<<<<<<
39392930 ttttcggtagtcctgaaaccagggctactgcctgtgctccaggtgcttgt 39392881

00001101 ttacctcttgaggtggccaagcatatgacatccttgggcctagcgtgcac 00001150
<<<<<<<< ||||||||||||||||||||||||||||||||||||||||||| |||||| <<<<<<<<
39392880 ttacctcttgaggtggccaagcatatgacatccttgggcctagtgtgcac 39392831

00001151 cataactgcttactagctgtgttaattccttgaatttctttagagcaagc 00001200
<<<<<<<< |||||||||||||||||||||||||||||||||||||||||||||||||| <<<<<<<<
39392830 cataactgcttactagctgtgttaattccttgaatttctttagagcaagc 39392781

00001201 aaaatctttttgaacacagttccagcttcagctttggccctgattccttc 00001250
<<<<<<<< |||||||||||||||||||||||||||||||||||||||||||||||||| <<<<<<<<
39392780 aaaatctttttgaacacagttccagcttcagctttggccctgattccttc 39392731

00001251 tcttttgccaagagcaactgctgtccccatacgcagccatcaatgagaag 00001300
<<<<<<<< |||||||||||||||||||||||||||||||||||||||||||||||||| <<<<<<<<
39392730 tcttttgccaagagcaactgctgtccccatacgcagccatcaatgagaag 39392681

00001301 gcttcaggcctgtctgtgctgtccctcaagacctttctctaggttgtaca 00001350
<<<<<<<< |||||||||||||||||||| ||||||||||||||||||||||||||||| <<<<<<<<
39392680 gcttcaggcctgtctgtgctatccctcaagacctttctctaggttgtaca 39392631

00001351 catacaagtgtggcacagggcatcatccagtgttctcatgtttttccttc 00001400
<<<<<<<< |||||||||||||||||||||||||||||||||||||||||||||||||| <<<<<<<<
39392630 catacaagtgtggcacagggcatcatccagtgttctcatgtttttccttc 39392581
```

```
00001401 agactgttgttaatttttcactgctctccctgcatttcatagggtgaatc 00001450
<<<<<<<< |||||||||||||||||||||||||||||||||||||||||||||||||| <<<<<<<<
39392580 agactgttgttaatttttcactgctctccctgcatttcatagggtgaatc 39392531

00001451 tatagagctgtatgctacactgctgcagtgtcacccacattgatgctact 00001500
<<<<<<<< |||||||||||||||||||||||||||||||||||||||||||||||||| <<<<<<<<
39392530 tatagagctgtatgctacactgctgcagtgtcacccacattgatgctact 39392481

00001501 agcaggcgtcagagttccacggccaggagatgctaaagtgcccttctcag 00001550
<<<<<<<< |||||||||||||||||||||||||||||||| | ||||||||||||||| <<<<<<<<
39392480 agcaggcgtcagagttccacggccaggagatgttgaagtgcccttctcag 39392431

00001551 cacttccagtgtcatgtcaaaaatcttgaaacttgtttccctttctggaa 00001600
<<<<<<<< |||||||||||||||||||||||||||||||||||||||||||||||||| <<<<<<<<
39392430 cacttccagtgtcatgtcaaaaatcttgaaacttgtttccctttctggaa 39392381

00001601 ggacctgaaattatttgtgaagattctttagaccatgtctatcaatctcc 00001650
<<<<<<<< |||||||||||||||||||||||||||| |||||||||||||||||||| <<<<<<<<
39392380 ggacctgaaattatttgtgaagattcttcagaccatgtctatcaatctcc 39392331

00001651 tgcttctcacataggcctgggcctttcctcagccaaggtcatcccca 00001697
<<<<<<<< ||||||||||||||||||||||||||||||||||||||||||||||| <<<<<<<<
39392330 tgcttctcacataggcctgggcctttcctcagccaaggtcatcccca 39392284

00001710 gggggaaatttctgagaaaacagtcatgagattccatttaaaagcaccct 00001759
<<<<<<<< |||||||||||||||||||||||||||||||||||||||||||||||||| <<<<<<<<
39392283 gggggaaatttctgagaaaacagtcatgagattccatttaaaagcaccct 39392234

00001760 agctgctttcacttcctttgctgtgtctgtttggagtgcgagtacagcct 00001809
<<<<<<<< |||||||||||||||||||||||||||||||||||||||||||||||||| <<<<<<<<
39392233 agctgctttcacttcctttgctgtgtctgtttggagtgcgagtacagcct 39392184

00001810 gcagagctttaaagacactggccctgccatccaaaactgaaacagtgccc 00001859
<<<<<<<< |||||||||||||||||||||||||||||||||||||||||||||||||| <<<<<<<<
39392183 gcagagctttaaagacactggccctgccatccaaaactgaaacagtgccc 39392134

00001860 tcacctactaaaaggataatccagttccaatctcttcctgttattttgat 00001909
<<<<<<<< |||||||||||||||||||||||||||||||||||||||||||||||||| <<<<<<<<
39392133 tcacctactaaaaggataatccagttccaatctcttcctgttattttgat 39392084

00001910 atggcctgtggttttgatcaaattgctgtgaccagcacttaggaaggctt 00001959
<<<<<<<< |||||||||||||||||||||||||||||||||||||||||||||||||| <<<<<<<<
39392083 atggcctgtggttttgatcaaattgctgtgaccagcacttaggaaggctt 39392034
```

```
00001960 tacagctcttggcacaatctgacacgacatgaacttgcaattaagagcac 00002009
<<<<<<<< |||||||||||||||||||||||||||||||||||||||||||||||||| <<<<<<<<
39392033 tacagctcttggcacaatctgacacgacatgaacttgcaattaagagcac 39391984

00002010 atgctctgctgaagctcagaatggggcctgtggtgagaaggggttcctaa 00002059
<<<<<<<< |||||||||||||||||||||||||||||||||||||||||||||||||| <<<<<<<<
39391983 atgctctgctgaagctcagaatggggcctgtggtgagaaggggttcctaa 39391934

00002060 agcacttcaaaatctcttcaaagctaagtgccagaatgctttattctatg 00002109
<<<<<<<< |||||||||||||||||||||||||||||||||||||||||||||||||| <<<<<<<<
39391933 agcacttcaaaatctcttcaaagctaagtgccagaatgctttattctatg 39391884

00002110 tagtgccaaattgtgagagggcagctctggatgtattaggaggaatttat 00002159
<<<<<<<< |||||||||||||||||||||||||||||||||||||||||||||||||| <<<<<<<<
39391883 tagtgccaaattgtgagagggcagctctggatgtattaggaggaatttat 39391834

00002160 tttgacactgacattaaattgagatgcccacttactctctacatgagcag 00002209
<<<<<<<< |||||||||||||||||||||||||||||||||||||||||||||||||| <<<<<<<<
39391833 tttgacactgacattaaattgagatgcccacttactctctacatgagcag 39391784

00002210 tgggcacctaaacttccttagtagtcactgaagctcggagagcaatggca 00002259
<<<<<<<< ||||||||||||||||||||||||||||||||||| |||||||||||||| <<<<<<<<
39391783 tgggcacctaaacttccttagtagtcactgaagcttggagagcaatggca 39391734

00002260 ttcccaatccacacaccactatgtagcctgctctatctgtagtggccaca 00002309
<<<<<<<< ||||||||||||||||| ||||||||||||||| |||||||||||||||| <<<<<<<<
39391733 ttcccaatccacacaccgctatgtagcctgctccatctgtagtggccaca 39391684

00002310 tctttcttgatatacttgggattatacatcaggtgcatttatatacacat 00002359
<<<<<<<< |||||||||||||||||||||||||||||||||||||||||||||||||| <<<<<<<<
39391683 tctttcttgatatacttgggattatacatcaggtgcatttatatacacat 39391634

00002360 gtatgtggatatctatagtcagatgtcttaaactgaatgcccaca..cat 00002407
<<<<<<<< |||||||||||||||||||||||||||||||||||||||||||||  ||| <<<<<<<<
39391633 gtatgtggatatctatagtcagatgtcttaaactgaatgcccacactcat 39391584

00002408 tacaaactgaacttgattttgcagcattcaaaagcacaggtgttttttt 00002457
<<<<<<<< |||||||||||||||||||||||||||||||||| ||||||||||||||| <<<<<<<<
39391583 tacaaactgaacttgattttgcagcattcaaaagtacaggtgttttttt 39391534

00002458 tttaagaaaggctattccagatcatctttgaaaactcgcttcagttctat 00002507
<<<<<<<< |||||||||||||||||||||||||||||||||||||||||||||||||| <<<<<<<<
39391533 tttaagaaaggctattccagatcatctttgaaaactcgcttcagttctat 39391484
```

```
00002508 gccatcaatatgtagccatatcagaaagccagtcctcttccactgtatta 00002557
<<<<<<<< |||||||||||||||||||||||||||||||||||||||||||||||||| <<<<<<<<
39391483 gccatcaatatgtagccatatcagaaagccagtcctcttccactgtatta 39391434

00002558 aaaacatttcttaagagagtatcatagaatagtttgggttggaagggacc 00002607
<<<<<<<< |||||||||||||||||||||||||||||||||||||||||||||||||| <<<<<<<<
39391433 aaaacatttcttaagagagtatcatagaatagtttgggttggaagggacc 39391384

00002608 tttaagatatctggttccagccccctgctataggcagggacacctccctc 00002657
<<<<<<<< |||||||||||||||||||||||||||| ||||||||||||||||||||| <<<<<<<<
39391383 tttaagatatctggttccagccccctgccataggcagggacacctccctc 39391334

00002658 tagaccaggctgctcacagccccatccagcctggcctggaatgcttccag 00002707
<<<<<<<< ||||||||||||||||||||||||||||||||||||| |||||||||||| <<<<<<<<
39391333 tagaccaggctgctcacagccccatccagcctggccttgaatgcttccag 39391284

00002708 ggaggggcatcaagctttgctgggaaagattttaactttttaaaagattt 00002757
<<<<<<<< ||||||||||||||| |||||||||||||||||| ||||||||||||||| <<<<<<<<
39391283 ggaggggcatcaagccttgctgggaaagattttatctttttaaaagattt 39391234

00002758 taatactttaaagattttaaacagaaatcataagtagaatttaaatattt 00002807
<<<<<<<< |||||||||||||||||||||||||||||||||||||||||||||||||| <<<<<<<<
39391233 taatactttaaagattttaaacagaaatcataagtagaatttaaatattt 39391184

00002808 ttagtaattagtttaatacctttgggaacattcataactgtagcatctgg 00002857
<<<<<<<< |||||||||||||||||||||||||||||||||||||||||||||||||| <<<<<<<<
39391183 ttagtaattagtttaatacctttgggaacattcataactgtagcatctgg 39391134

00002858 acactcaaaaccactccagttcagctccagctttaactaagaagtggaaa 00002907
<<<<<<<< |||||||||||||||||||||||||||||||||||||||||||||||||| <<<<<<<<
39391133 acactcaaaaccactccagttcagctccagctttaactaagaagtggaaa 39391084

00002908 acttccaaagagatgcattacaatgagctctatcagaacatccaggttga 00002957
<<<<<<<< |||||||||||||||||||||||||||||||||||||||||||||||||| <<<<<<<<
39391083 acttccaaagagatgcattacaatgagctctatcagaacatccaggttga 39391034

00002958 aatatcttcctttttcattatcagaaatggatctgcagtcaggcagtttc 00003007
<<<<<<<< |||||||||||||||||||||||||||||||||||||||||||||||||| <<<<<<<<
39391033 aatatcttcctttttcattatcagaaatggatctgcagtcaggcagtttc 39390984

00003008 cacagctccatatctagatatacaggaaaaaacatttgcattcagttttt 00003057
<<<<<<<< |||||||||||||||||||||||||||||||||||||||||||||||||| <<<<<<<<
39390983 cacagctccatatctagatatacaggaaaaaacatttgcattcagttttt 39390934
```

```
00003058 agcctgttgtattctgtagttgattttgcattattacaactgggtagaag 00003107
<<<<<<<< |||||||||||||||||||||||||||||||||||||||||||||||||| <<<<<<<<
39390933 agcctgttgtattctgtagttgattttgcattattacaactgggtagaag 39390884

00003108 aaagaaacatggcaaagtgtttggaaatatttgagtgcccttcatgttga 00003157
<<<<<<<< || |||||||||||||| |||||||||||||||||||||||||||||||| <<<<<<<<
39390883 aatgaaacatggcaaagcgtttggaaatatttgagtgcccttcatgttga 39390834

00003158 caaagaatgagaaatattcttattgctcagtaatctttgctgtctgattc 00003207
<<<<<<<< |||||||||||||||||||||||||||||||||||||||||||||||||| <<<<<<<<
39390833 caaagaatgagaaatattcttattgctcagtaatctttgctgtctgattc 39390784

00003208 tgaagacctgcatctcttctacatctgctttcctcgggattcctccccac 00003257
<<<<<<<< ||||||||||||||||||||||||||||||||||||||| |||||||||| <<<<<<<<
39390783 tgaagacctgcatctcttctacatctgctttcctcgggaatcctccccac 39390734

00003258 agcccttcagtcaatactcctggctcactctctactttcttcgtcttcca 00003307
<<<<<<<< |||||||||||||||||||||||||||||||||||||||||||||||||| <<<<<<<<
39390733 agcccttcagtcaatactcctggctcactctctactttcttcgtcttcca 39390684

00003308 caaagtggagctacagctgcactgagagaaagggatgcggtgaagtgatt 00003357
<<<<<<<< |||||||||||||||||||||||||||||||||||||||||||||||||| <<<<<<<<
39390683 caaagtggagctacagctgcactgagagaaagggatgcggtgaagtgatt 39390634

00003358 gaagcataaggaagcagcactctgcacactgcaaaaagttgaaacttagc 00003407
<<<<<<<< |||||||||||||||||||||||||||| ||||||||||||||||||||| <<<<<<<<
39390633 gaagcataaggaagcagcactctgcacattgcaaaaagttgaaacttagc 39390584

00003408 agttgcatgtgaaggagagccagttcactaacccggctctgcagtgctct 00003457
<<<<<<<< |||||||||||||||||||||||||||||||||||||||||||||||||| <<<<<<<<
39390583 agttgcatgtgaaggagagccagttcactaacccggctctgcagtgctct 39390534

00003458 aggcccagaaattaggcttctcaatccatcttgactgagcagggaagtgc 00003507
<<<<<<<< |||||||||||||||||||||||||||||||||||||||||||||||||| <<<<<<<<
39390533 aggcccagaaattaggcttctcaatccatcttgactgagcagggaagtgc 39390484

00003508 ttgtacgtggcacacctct.....accttggcacaaatctgatctgagtc 00003552
<<<<<<<< |||||||||||||||||||     |||||||||||||||||||||||||| <<<<<<<<
39390483 ttgtacgtggcacacctcttcctcaccttggcacaaatctgatctgagtc 39390434

00003553 tggccaggatatgtttcttgggaaaacatctgttacttttccaatcagtg 00003602
<<<<<<<< |||||||||||||||||||||||||||||||||||||||||||||||||| <<<<<<<<
39390433 tggccaggatatgtttcttgggaaaacatctgttacttttccaatcagtg 39390384
```

00003603 gttacacttacaggtagaaaacaaccactatgatgctcatctctagcgtg 00003652
<<<<<<<< |||||||||||||||||||||||||||||||||||||||||||||||||| <<<<<<<<
39390383 gttacacttacaggtagaaaacaaccactatgatgctcatctctagcgtg 39390334

00003653 gaagagcgtggcagttttagtctcttccctgggcaagttcagatatactt 00003702
<<<<<<<< |||||||||||||||||||||||||||||||||||||||||||||||||| <<<<<<<<
39390333 gaagagcgtggcagttttagtctcttccctgggcaagttcagatatactt 39390284

00003703 tcatagttttgttcagctctctgagaaagatctaaatgcatggtgatgtg 00003752
<<<<<<<< |||||||||||||||||||||||||||||||||||||||||||||||||| <<<<<<<<
39390283 tcatagttttgttcagctctctgagaaagatctaaatgcatggtgatgtg 39390234

00003753 tgaggggaggcgagtagcttcagtgtgatgccccacagttctgcataagg 00003802
<<<<<<<< |||||||||||||||||||||||||||||||||||||||||||||||||| <<<<<<<<
39390233 tgaggggaggcgagtagcttcagtgtgatgccccacagttctgcataagg 39390184

00003803 agttctgtgaaatctctgaaaactattctgtgataccttactgctgagtg 00003852
<<<<<<<< |||||||||||||||||||||||||||||||||||||||||||||||||| <<<<<<<<
39390183 agttctgtgaaatctctgaaaactattctgtgataccttactgctgagtg 39390134

00003853 agagcatgcaagtagtcctggggctggagttgggccaggaccaaagctct 00003902
<<<<<<<< |||||||||||||||||||||||||||||||||||| ||||||||||||| <<<<<<<<
39390133 agagcatgcaagtagtcctggggctggagttgggccgggaccaaagctct 39390084

00003903 aagcactgctgcagccctgtgtggaatataacttatctgaagccaaatta 00003952
<<<<<<<< |||||||||||||||||||||||||||||||||||||||||||||||||| <<<<<<<<
39390083 aagcactgctgcagccctgtgtggaatataacttatctgaagccaaatta 39390034

00003953 gtggcattcgctttctgcctacaggagaggagtaatactgcttgctaact 00004002
<<<<<<<< |||||||||||||||||||||||||||||||||||||||||||||||||| <<<<<<<<
39390033 gtggcattcgctttctgcctacaggagaggagtaatactgcttgctaact 39389984

00004003 cctgactgacgcaatccttgagatacaatggtcaggatttatacagtgcc 00004052
<<<<<<<< |||||||||||||||||||||||||||||||||||||||||||||||||| <<<<<<<<
39389983 cctgactgacgcaatccttgagatacaatggtcaggatttatacagtgcc 39389934

00004053 ttgaaaaaccaaggagtcatataaatcctaattgggtattaatggtgttg 00004102
<<<<<<<< ||||||||||||||||||||||||||||||||||||||||||||| || <<<<<<<<
39389933 ttgaaaaaccaaggagtcatataaatcctaattgggtattaatgg...tg 39389887

00004103 ttattatataactatggtacatgctacctacaacactttttttgtcagtt 00004152
<<<<<<<< |||||||||||||||||||||||||||||||||||||||||||||||||| <<<<<<<<
39389886 ttattatataactatggtacatgctacctacaacactttttttgtcagtt 39389837

```
00004153 tacttattattgtttgctcattacatgtgtggaaatagaggtagcagaaa 00004202
<<<<<<<< |||||||||||||||||||||||||||||||||||||||||||||||||| <<<<<<<<
39389836 tacttattattgtttgctcattacatgtgtggaaatagaggtagcagaaa 39389787

00004203 catgaagtaaattatatgagattccctatttaaaaaaaaatctatttgtc 00004252
<<<<<<<< |||||||||||||||||||||||||||||||||||||||||||||||||| <<<<<<<<
39389786 catgaagtaaattatatgagattccctatttaaaaaaaaatctatttgtc 39389737

00004253 agagtcaggagtaaaacttgatcatcccaatgtagcattctgcccatcag 00004302
<<<<<<<< |||||||||||||||||||||||||||||||||||||||||||||||||| <<<<<<<<
39389736 agagtcaggagtaaaacttgatcatcccaatgtagcattctgcccatcag 39389687

00004303 gccagccattacttaattagctcctaaatacataagaagtccaattaact 00004352
<<<<<<<< |||||||||||||||||||||||||||||||||||||||||||||||||| <<<<<<<<
39389686 gccagccattacttaattagctcctaaatacataagaagtccaattaact 39389637

00004353 aggatagtgagcctgacagaaaaaacaacagctgcagctgtggttggaat 00004402
<<<<<<<< |||||||||||||||||||||||||||||||||||||||||||||||||| <<<<<<<<
39389636 aggatagtgagcctgacagaaaaaacaacagctgcagctgtggttggaat 39389587

00004403 cagaaaaggtttgggaaatgcagtcctgggttgctgagtaggaaagaaaa 00004452
<<<<<<<< |||| |||||||||||||||||||||||||||| |||||||||||||||| <<<<<<<<
39389586 cagagaaggtttgggaaatgcagtcctgggttgttgagtaggaaagaaaa 39389537

00004453 cagtgtacatgtagctgcaaggtagcagtactgccagtggtgtgatttgg 00004502
<<<<<<<< |||||||||||||||||||||||||||||||||||||||||||||||||| <<<<<<<<
39389536 cagtgtacatgtagctgcaaggtagcagtactgccagtggtgtgatttgg 39389487

00004503 aaacatattaaggcagtgattcacccactttctatctacaggatcagtgt 00004552
<<<<<<<< |||||||||||||||||||||||||||||||||||||||||||||||||| <<<<<<<<
39389486 aaacatattaaggcagtgattcacccactttctatctacaggatcagtgt 39389437

00004553 ggttcagaagatggatcaaccacgtctgtgtctgaacagaccctcattga 00004602
<<<<<<<< |||| ||||||||||||||||||||||||||||||||||||||||||||| <<<<<<<<
39389436 ggtttagaagatggatcaaccacgtctgtgtctgaacagaccctcattga 39389387

00004603 tctgaaggtgagaagctcatggaggttaagttgggtgagacagtggctga 00004652
<<<<<<<< |||||||||||||||||||||||||||||||||||||||||||||||||| <<<<<<<<
39389386 tctgaaggtgagaagctcatggaggttaagttgggtgagacagtggctga 39389337

00004653 tgggtcattggcctaatgggactgacgctgaacgtccctgaggtacagat 00004702
<<<<<<<< |||||||||||||||||||||||||||||||||||||||||||||||||| <<<<<<<<
39389336 tgggtcattggcctaatgggactgacgctgaacgtccctgaggtacagat 39389287
```
